# The effect of charge-balanced transcutaneous electrical nerve stimulation on rodent facial nerve regeneration

**DOI:** 10.1038/s41598-022-05542-y

**Published:** 2022-01-26

**Authors:** Young Sang Cho, Onjeon Ryu, Kyeongwon Cho, Dohyoung Kim, Jihyun Lim, Sung Hwa Hong, Yang-Sun Cho

**Affiliations:** 1 Department of Otorhinolaryngology-Head and Neck Surgery, Samsung Medical Center, Sungkyunkwan University School of Medicine, Seoul, Korea; 2 NuEyne Co., Ltd., Seoul, Korea; 3 Center for Clinical Epidemiology, Samsung Medical Center, Seoul, Korea; 4 Department of Otorhinolaryngology-Head and Neck Surgery, Samsung Changwon Hospital, Sungkyunkwan University School of Medicine, Changwon, Korea

**Keywords:** Neurogenesis, Peripheral nervous system, Neural tube defects, Electrical and electronic engineering

## Abstract

This study aimed to investigate the effect of charge-balanced transcutaneous electrical nerve stimulation (cb-TENS) in accelerating recovery of the facial function and nerve regeneration after facial nerve (FN) section in a rat model. The main trunk of the left FN was divided and immediately sutured just distal to the stylomastoid foramen in 66 Sprague–Dawley rats. The control group had no electrical stimulus. The other two groups received cb-TENS at 20 Hz (20 Hz group) or 40 Hz (40 Hz group). Cb-TENS was administered daily for seven days and then twice a week for three weeks thereafter. To assess the recovery of facial function, whisker movement was monitored for four weeks. Histopathological evaluation of nerve regeneration was performed using transmission electron microscopy (TEM) and confocal microscopy with immunofluorescence (IF) staining. In addition, the levels of various molecular biological markers that affect nerve regeneration were analyzed. Whisker movement in the cb-TENS groups showed faster and better recovery than the control group. The 40 Hz group showed significantly better movement at the first week after injury (*p* < 0.0125). In histopathological analyses using TEM, nerve axons and Schwann cells, which were destroyed immediately after the injury, recovered in all groups over time. However, the regeneration of the myelin sheath was remarkably rapid and thicker in the 20 Hz and 40 Hz groups than in the control group. Image analysis using IF staining showed that the expression levels of S100B and NF200 increased over time in all groups. Specifically, the expression of NF200 in the 20 Hz and 40 Hz groups increased markedly compared to the control group. The real-time polymerase chain reaction was performed on ten representative neurotrophic factors, and the levels of IL-1β and IL-6 were significantly higher in the 20 and 40 Hz groups than in the control group (*p* < 0.015). Cb-TENS facilitated and accelerated FN recovery in the rat model, as it significantly reduced the recovery time for the whisker movement. The histopathological study and analysis of neurotrophic factors supported the role of cb-TENS in the enhanced regeneration of the FN.

## Introduction

Facial nerve palsy (FNP) is not only a defect in the functioning of facial muscles and expression, but also a possible cause of a severe emotional and psychological problem with delayed or incomplete recovery. With FNP, individuals' nonverbal facial signs are often misinterpreted, making social life challenging. Moreover, there are many problems and limitations in chewing, drinking, and ophthalmological functions^1,2^.

Bell's palsy (acute idiopathic facial palsy) is the most common cause of FNP, but it can also be caused by trauma, inflammation, tumors, or surgical procedures^3^. The rate of axonal regeneration determines the degree of functional recovery, but there is wide variation in the recovery rates and final outcomes^4^.

There are various treatment options for FNP depending on its cause and duration. In Bell's palsy, which accounts for over 50% of all FNP, corticosteroids or antiviral drugs are the primary treatment. The use of corticosteroids has shown significant benefits in many randomized controlled trials^5^, but the evidence for antiviral agents is insufficient^6^. Other treatment options for facial nerve (FN) damage include surgical repair and low-frequency electrical stimulation^7^. However, severe FN damage usually results in incomplete recovery of House-Brackmann (H-B) grade III-IV even after an appropriate treatment^8,9^. Therefore, treatments that accelerate facial function recovery and improve the final outcomes are still in demand.

Peripheral motor nerves, such as the FN, can regenerate axons. However, the degree of recovery varies^10^. In general, damage to nerve tissues first causes a local inflammatory reaction regulated by numerous signaling molecules, including cytokines. The production of cytokines and local inflammation can affect the outcome of neurological recovery^11^. In particular, the initial response to the injury when the Wallerian degeneration progresses is highly important for the physiological and functional recovery of nerves^12^.

Electrical stimulation (ES) is a potential method that has been actively explored for neuronal damage and recovery^3^. To date, many studies have demonstrated that low-frequency ES has functional and molecular effects on nerve regeneration in animal experiments^3,10,13,14^. Most results showed a modest degree of FN regeneration and functional recovery. However, these studies employed an invasive insertion of electrodes to provide ES, which makes its clinical use challenging. Transcutaneous electrical nerve stimulation (TENS), on the other hand, is more practical in clinical applications as it is non-invasive, easy to handle, and can be used for a long time. Current studies involving TENS mostly examined its analgesic and muscle-relaxing effects^15^, and only a few studies investigated the effects on nerve regeneration including that of the FN^16,17^.

Many studies on peripheral nerve regeneration used electrical stimulation with constant or monophasic electrical currents to acquire a more direct effect of the electrical current. However, they did not consider an electrical charge balance, and tissue injury was subjected to occur. In a recent study, electrical charge balancing by using the biphasic pulse was used to prevent potential damage to the affected tissue while the stimulation frequency was transmitted^18^. Our study used a charge-balanced (cb) current stimulation method with a biphasic electric pulse signal as a mirror-image pattern to prevent electric charges from accumulating in tissues or cells^19^.

In the present study, the FNs in rats were injured, repaired, and treated with cb-TENS. The effects of ES on functional recovery, molecular marker expression, and histological findings were investigated.

## Results

### Functional recovery of whisker movement

**Figure 1:**
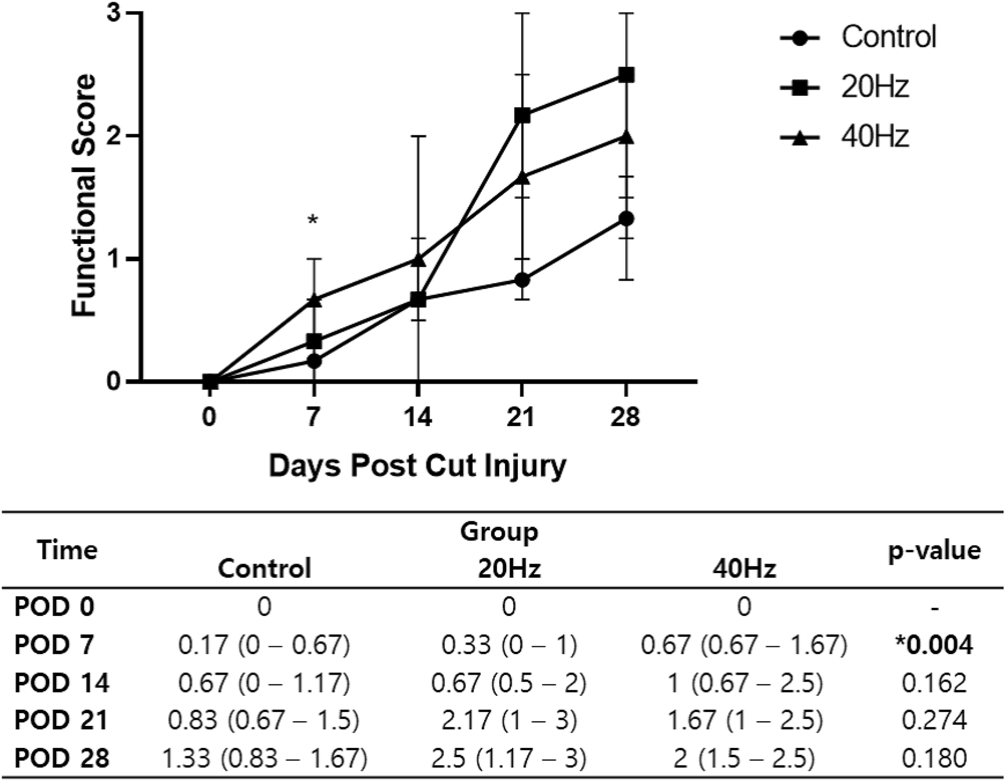
Functional score analyzed through whisker movement. Three independent observers evaluated the whisker movement with the following scale: 0 (no detectable movement), 1 (detectable motion), 2 (significant but asymmetric voluntary motion), and 3 (symmetric voluntary motion). The median (range) values are shown in the figure. Considering multiple comparisons, *p < 0.0125 is significant.

Three blinded observers analyzed whisker movement four times on PODs 0, 7, 14, 21, and 28. The whisker movement improved over time in all rats, including the control group that did not receive any stimulation. However, for the groups that received the cb-TENS treatments, both the 20-Hz and the 40-Hz stimuli were superior to the control group in terms of the rate and degree of recovery. As a result, statistical significance was only observed in the 40 Hz group (*p* < 0.0125, the Kruskal–Wallis H test) on POD 7 (Fig. 1). All rats had a score of 0 immediately after the FN injury, but in the control group, the median score was 0.17 on POD 7 and 1.33 on POD 28. The 20 Hz and the 40 Hz groups showed median scores of 0.33 and 0.67 points on POD 7 and 2.5 and 2.0 points on POD 28, respectively.

### Molecular analysis

**Figure 2:**
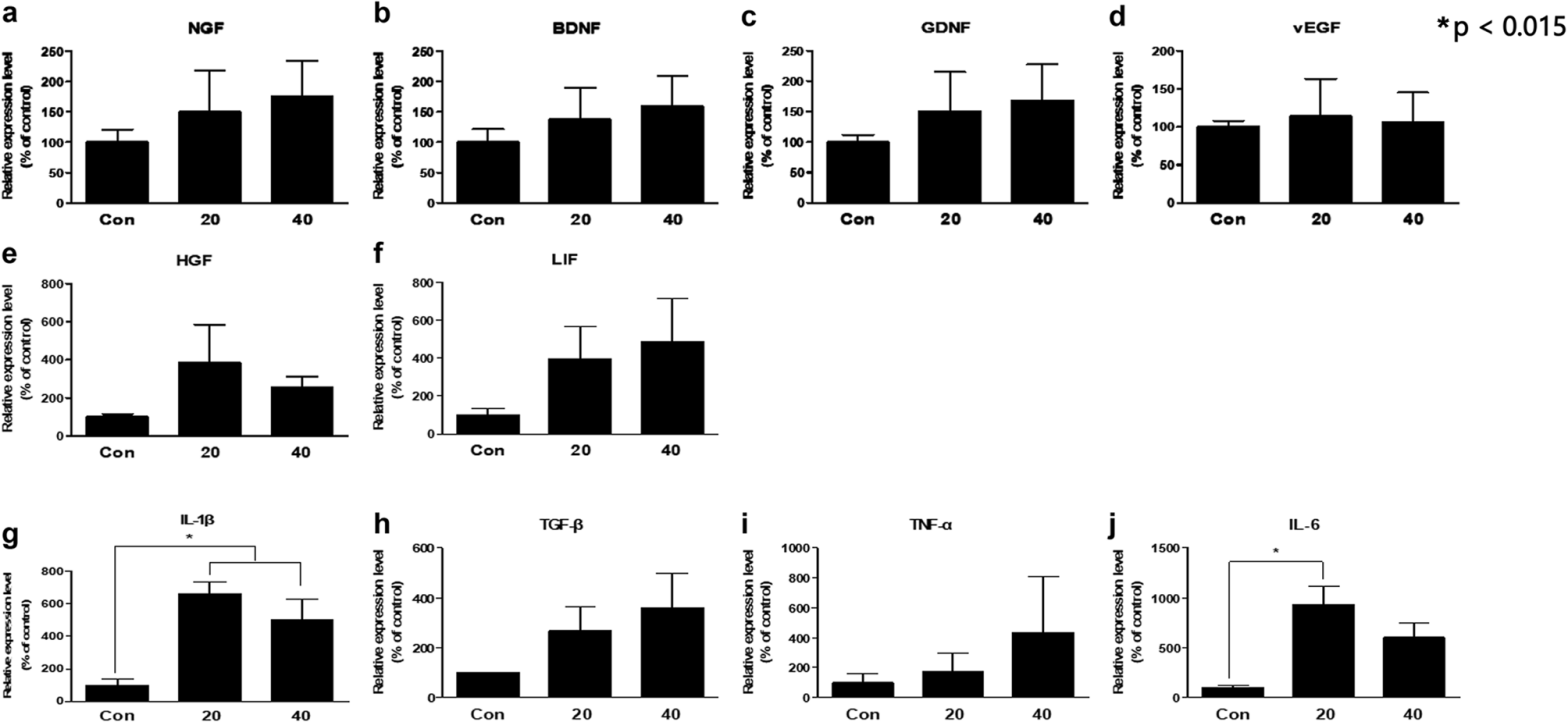
Molecular analysis results. Results of analyzing ten neurotrophic factors using real-time quantitative polymerase chain reaction. (**a**) Nerve growth factor (NGF), (**b**) Brain-derived neurotrophic factor (BDNF), (**c**) Glial cell-derived neurotrophic factor (GDNF). (**d**) Vascular endothelial growth factor (VEGF), (**e**) Hepatocyte growth factor (HGF), (**f**) Leukemia inhibitory factor (LIF), (**g**) Interleukin (IL-1β), (**h**) Transforming growth factor-β (TGF-β), (**i**) Tumor necrosis factor-α (TNF-α), (**j**) Interleukin-6 (IL-6). *p < 0.015 is significant.

Molecular analysis was performed on POD 28. For this analysis, three rats were selected randomly for each group. Real time polymerase chain reaction (RT-PCR) was completed for ten molecular markers affecting neural regeneration. Although not statistically significant, the levels of all molecules involved in nerve growth (NGF, BDNF), angiogenesis (VEGF), cell proliferation (LIF, HGF), nerve reinnervation (GDNF), and nerve regeneration (IL-1β, IL-6, TNF-α, TGF-β) were higher in the cb-TENS groups than in the control group (Fig. 2, the Tukey's test). In particular, the levels of pro-inflammatory cytokines IL-1β and IL-6 were significantly (*p* < 0.015) higher in the 20 Hz and 40 Hz groups than in the control group (Fig. 2g,j).

### Histopathological analysis

The distal part of the injured FN trunk was harvested, and histopathological analysis was conducted in two ways. First, the microstructures and myelin sheaths of axons at 5000 nm scope were analyzed using TEM. Second, a semi-quantitative analysis of NF200 and S100 proteins was performed using a fluorescence microscope. To observe pathological changes over time, samples for each group were obtained and analyzed on PODs 7, 14, and 28. For histopathological analysis, the results were compared to those of the rats with intact FNs (normal intact group).

**Figure 3:**
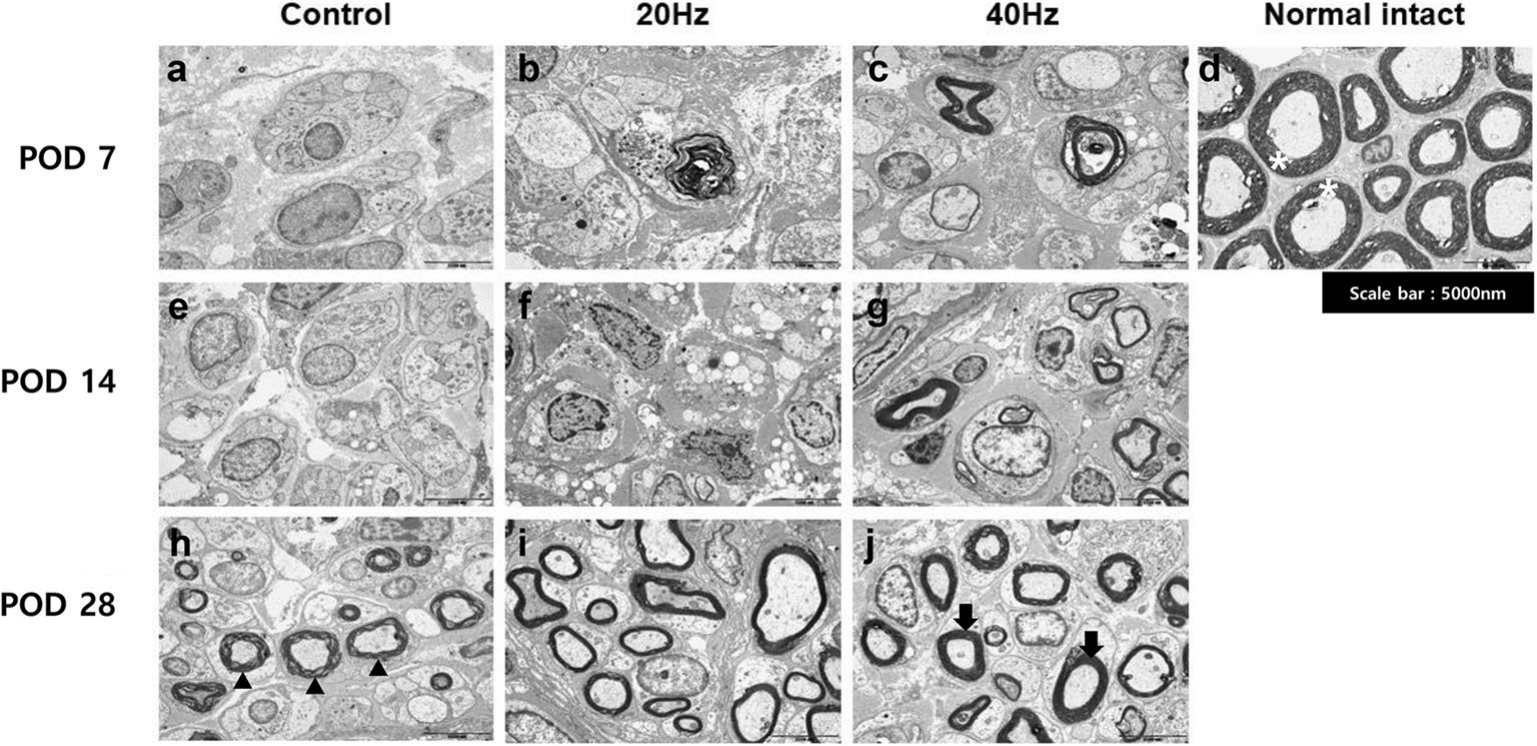
Cross-section transmission electron microscopy images of the facial nerves. Images of the 7th day (**a**–**c**), 14th day (**e**–**g**), and 28th day (**h**–**j**) after the facial nerve injury of each group and images of the normal intact group with no facial nerve damage (**d**). (*) Thick and homogeneous intact myelin sheath; (black triangle) disorganization of myelin sheath lamellae; (black arrow) well-organized myelin sheath lamellae.

For the rats with intact FNs, myelinated Schwann cells were observed to be remarkably intact (Fig. 3d). Schwann cells in all three groups (control, 40 Hz, and 20 Hz) were destroyed and axons also showed significant degeneration (Fig. 3a–c) at POD 7. At POD 14, there was no significant change from the first week in the control group (Fig. 3a,e), but regeneration of the myelin sheath was partially observed in the 20 Hz and 40 Hz groups (Fig. 3f,g). Although myelin sheath regeneration was observed in all groups at POD 28, the control group showed the presence of axonal degeneration, non-myelinated axons, and disorganization of myelin sheath lamellae (Fig. 3h). The 20 Hz and 40 Hz groups, on the other hand, showed well-organized lamellae (Fig. 3i,j).

**Figure 4:**
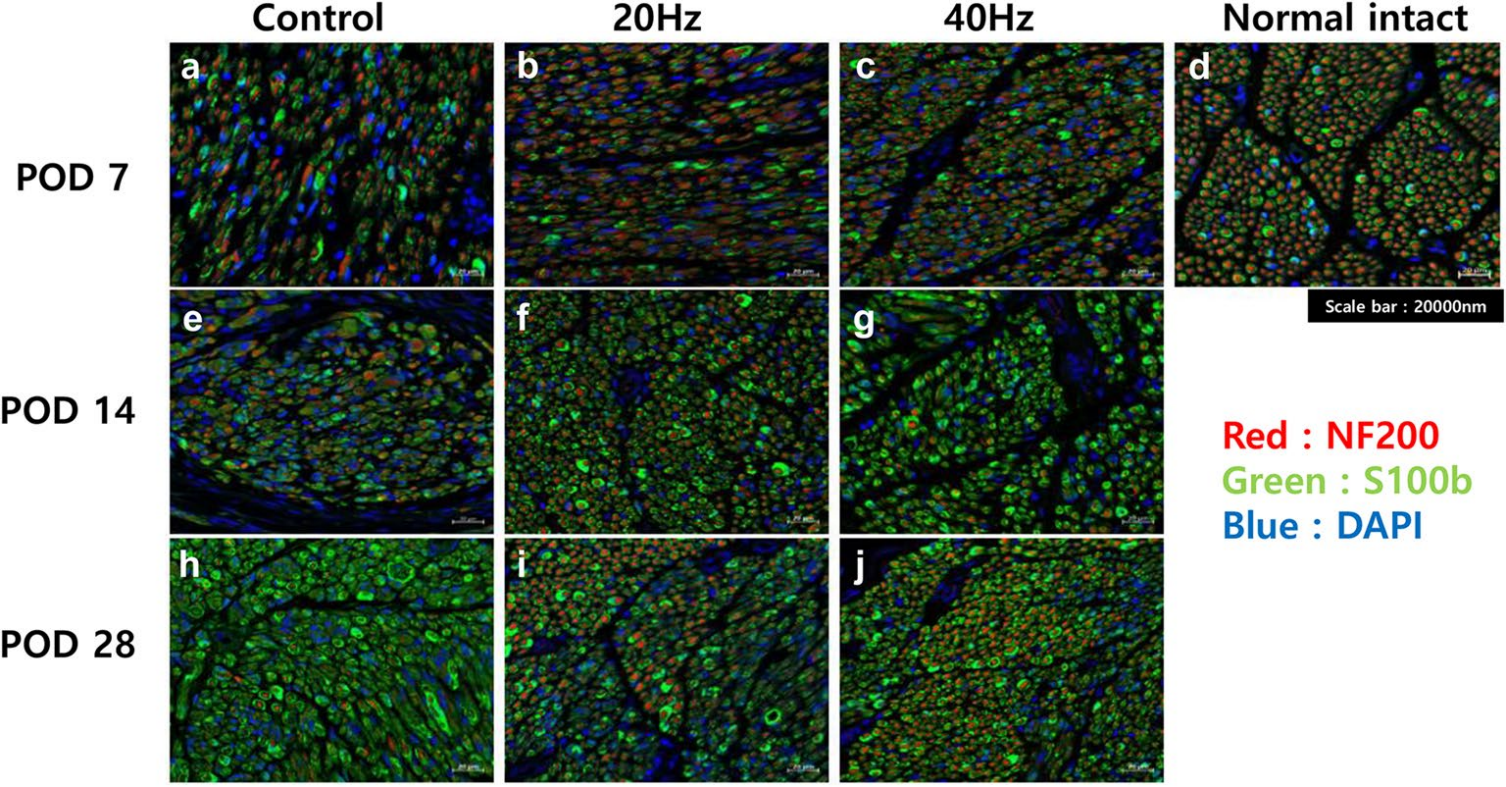
Expression levels of S100B and NF200 proteins in the distal portion of the injured facial nerves with immunofluorescence staining. Images of the 7th day (**a**–**c**), 14th day (**e**–**g**), and 28th day (**h**–**j**) after facial nerve injury of each group and images of normal intact rats with no facial nerve damage (**d**). Over time, the expression of S100B increased in all groups, but the expression of NF100 significantly increased in the 20 Hz and 40 Hz groups that received electrical stimulation.

**Figure 5:**
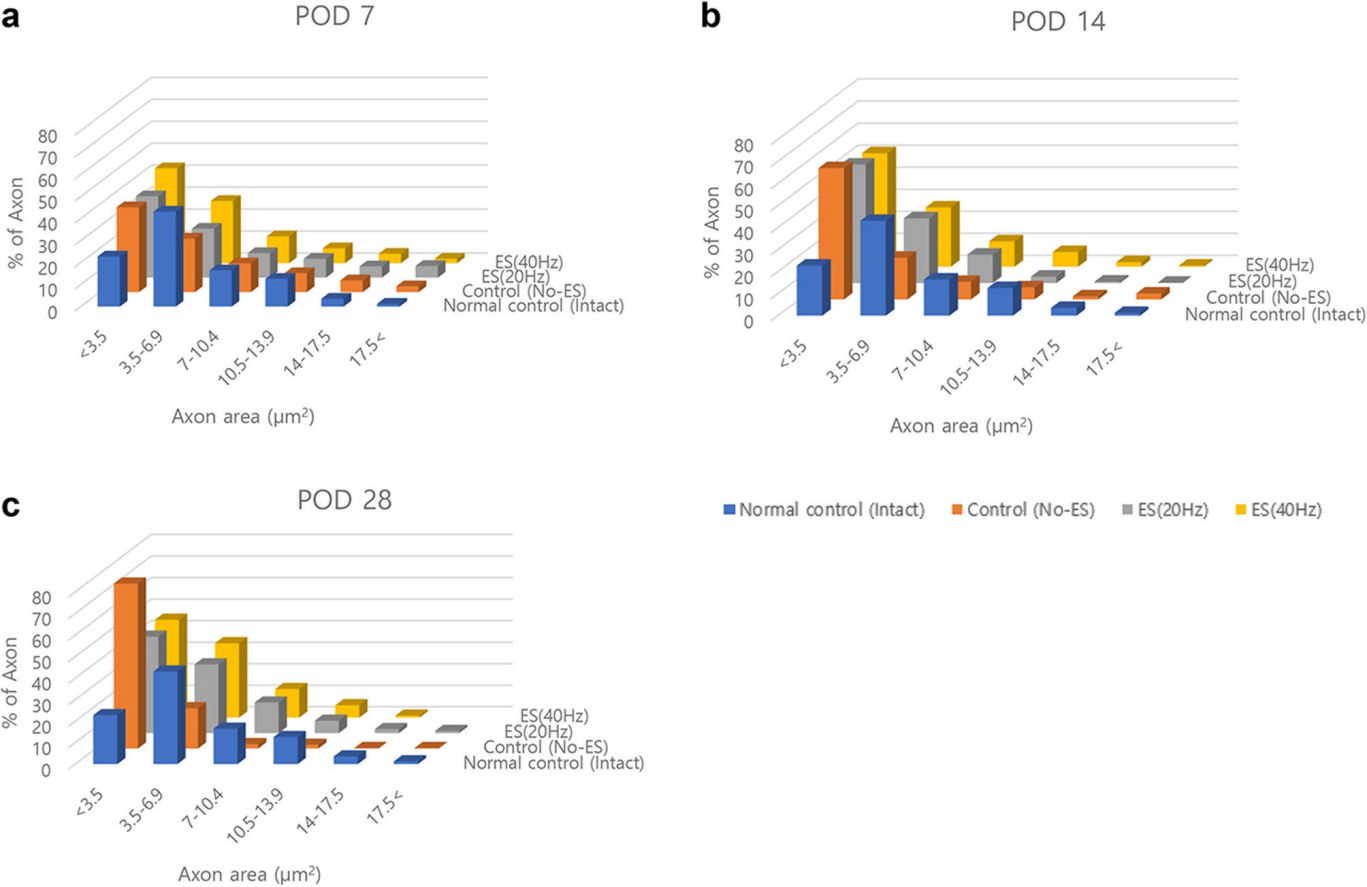
Changes over time in the areas of the axon filaments after facial nerve injury. In the normal intact group, the axon area was the largest in the range of 3.5 to 6.9 μm^2^, but the control, 20 Hz, and 40 Hz groups showed the largest proportion of axonal areas of 3.5 μm^2^ or less on the 7th day of injury (**a**). In particular, on the 14th day, the proportion of axon areas less than 3.5 μm^2^ was observed for all injury groups (**b**), but on the 28th day, the proportions of axon areas of 3.5 to 6.9 μm^2^ in the 20 Hz and 40 Hz groups increased, while the proportion of axon areas of 3.5 μm^2^ or less in the control group increased (**c**).

For further analysis, IF staining was performed on the specimens of each group (Fig. 4). The expression of S100B protein decreased significantly by POD 14 (Fig. 5e–g), but on POD 28, the expression of S100B had markedly increased regardless of the group. The expression levels of NF200 in the 20 Hz and 40 Hz groups (Fig. 4i,j) were markedly increased compared to that of the control group (Fig. 4h).

The FN axon fiber diameter was measured and analyzed with IF staining using ImageJ software (Fig. 5). The axon filaments were first divided into seven categories (< 3.5, 3.5–6.9, 7–10.4, 10.5–13.9, 14–17.5, and 17.5 <) based on their diameters expressed in square micrometers. Then, the percentage of the axon filaments for each group’s category was compared to the normal intact group. In the normal intact group, the area of the axon was observed to be mainly composed of a thickness of about 3.5 to 6.9 μm^2^. On POD 7, the axons were regenerated with a thickness of 3.5 μm^2^ or less in all groups with FN injuries. The proportion of axons with a thickness of 3.5 to 6.9 μm^2^ increased in the 20 Hz and 40 Hz groups over time, but not in the control group.

## Discussion

The present study demonstrated that cb-TENS could facilitate the regeneration of damaged FNs in the transection and surgical repair model. While a 20 Hz stimulus is known as the physiologically relevant frequency of motoneurons^20^, we also investigated the effects of a 40 Hz stimulus which is the nearest harmonic frequency of 20 Hz. Sharma et al*.* had confirmed the effects of 20 Hz electrical stimulation to enhance the expression of regeneration-associated genes and proteins; BDNF, α1-tubulin and the GAP-43 in rat models with crushed facial nerve^21^. However, in some studies, optimal effects of nerve regeneration were reported at a higher or lower frequency than 20Hz^22,23^. In the scope of neural engineering, the functionality of the low frequency stimulation, which was used in this study, might be related to damaged nerve regeneration with enhanced release of neurotrophins^24^.

There are also reports that TENS applied for more than 4 days can induce opioid tolerance^25^ and consequently decrease peripheral nerve regeneration^26^. Although we did not measure the amount of opioids or receptors in this study, the results were opposite in terms of neural regeneration. This is probably because, unlike previous studies, electrical stimulation was applied immediately after the nerve damage, and maintenance stimulation was applied less frequently (twice per week) compared to other studies. Also, there may be the effect of the charge-balanced pulse stimulation, which is different from the ordinary pulse stimulation.

Although there was a slight difference in the recovery rate and pattern based on the stimulation frequency, the groups that received the cb-TENS showed faster functional and histological recovery than the control group. And it can be argued that 20 Hz stimulation is more suitable for treatment than 40 Hz because relatively low frequency is more stable and consumes less battery. However, more research is needed on the effect of nerve regeneration on stimulation between 20 and 200 Hz.

Research on the effectiveness of ES in the regeneration of peripheral nerves first appeared in the 2000s. However, most studies have investigated its effectiveness using invasive insertion or wrapping electrodes in animal models^3,27,28^. Therefore, these stimulation methods have limitations when it comes to clinical applications. In addition, research to date has used the direct current stimulation method, which can cause some side effects by accumulating electric charges in tissues or cells^19^. Our study used cb-TENS with a biphasic electric pulse signal as a mirror-image pattern to overcome the shortcomings of direct current. This method can be widely used in a variety of clinical settings.

When the peripheral nerve is damaged, the axon distal to the damaged area undergoes a characteristic sequence of changes in Wallerian degeneration^29^. In these pathological processes, pro-inflammatory and anti-inflammatory cytokines are required^30^. At the beginning of Wallerian degeneration, macrophages remove debris from destroyed myelin and axons while simultaneously producing cytokines such as IL-1, IL-12, and TNF-α. These cytokines subsequently induce NGF, IL-6, and LIF production and regenerate the peripheral nerves by supporting the serial expression and recruitment of IL-4, IL-10, TNF-α, and IFN-γ^11^. ES facilitates the expressions of these cytokines, which have an important effect on the regeneration of the peripheral nerves and the recovery of their function^31–33^. These findings are consistent with previous in vitro experiments that depolarization increased the influx of calcium ions into cells, expressing various genes and promoting neurite overgrowth^34^. Therefore, ES that opens calcium ion channels can promote the regeneration of tissue including nerves, regardless of the mode of current transmission.

The authors demonstrated through a video analysis that cb-TENS facilitates functional recovery of the damaged FN. On POD 7, the 40 Hz group showed significant functional recovery. On POD 28, both 20 Hz and 40 Hz groups showed higher functional scores than the control group. However, there was no statistical significance, probably because the number of rats per group decreased over time and the control group also recovered relatively well over time. The rapid recovery of the cb-TENS group and the good recovery of the control group are thought to be due to the nerve anastomosis immediately after the cut injury. Therefore, although there was no statistical difference in the later part of the experiment, it can be suggested that the groups receiving cb-TENS recovered facial function faster and better than the control group, in accordance with previous studies using TENS^3,13^. However, our method has the advantage of being non-invasive and providing continuous stimulation.

As shown in Fig. 2, the 20 Hz and 40 Hz groups showed higher expression levels of the molecular markers than the control group, but statistically significant differences were found for IL-1β and IL-6. As mentioned above, these pro-inflammatory cytokines remove debris and help promote axon regeneration when nerve damage occurs. Monocyte chemoattractant protein-1 (MCP-1), TNF-α, IL-1β, IL-6, and LIF are mainly responsible for recruiting macrophages and other immune cells^35^. In the present study, it is difficult to clearly demonstrate the mechanisms of upregulation of these cytokines, but ES has probably influenced the ion channel opening by a similar mechanism of BDNF and tyrosine receptor kinase B (trkB) upregulation by opening the voltage-gated Ca2^+^ channel^31^. Likewise, it is a well-known that early genes related to nerve regeneration are expressed in large quantities due to the influx of calcium ions into the cell body^28^. Conversely, another report investigated the role of calcium ion influx in axonal degeneration^36^. The distal portion of the damaged nerve must be rapidly and effectively degenerated for rapid regeneration^12^, and the voltage-gated Ca^2+^ channels seem to play a critical role in both processes.

The degree of histopathological recovery was confirmed by TEM and IF staining. As shown in Fig. 3, the myelin sheath was thick and homogeneous, and the shape of a myelinated axon was round and relatively constant in the intact nerve without damage (Fig. 3d). The nuclei were enlarged in all groups, and normal Schwann cells or myelin sheaths were not observed on POD 7. On the 28th day, many myelinated axons appeared in each group. However, the control group (Fig. 3h) showed an irregular myelin sheath lamellae organization compared to the 20 Hz and 40 Hz groups (Fig. 3i–j).

Expressions of NF200 and S100B were confirmed through IF staining, and additional image analysis was performed to compare the area of axon filaments (NF200-positive) for each group. In addition, the distribution of axon filaments for each group was analyzed based on the thickness of each axon. Even after 28 days, axon filaments with thicknesses of less than 3.5 μm^2^ accounted for almost 80% of the total in the control group. However, in the 20 Hz and 40 Hz groups, more than half of the axons were regenerated with a thickness of 3.5 μm^2^ or more (Fig. 5c). This means that each axon filament regenerated more effectively in the group that received ES than in the group that did not.

This study has several limitations. First, the FN injury model was made with rats whose FNs were divided and immediately repaired. This model has a problem in that the rate of spontaneous recovery is too high, which requires another model of delayed repair or viral infection to better prove the effectiveness of an intervention. Second, the safety of all clinically applied devices should be thoroughly verified. Although no skin burns or other side effects were observed when ES was administered for 28 days, the stability for long-term use was not investigated. Therefore, further studies on adverse effects are needed after long-term use for more than three months in the preclinical research stage. Third, the analysis results of whisker movement and molecular markers showed a tendency to increase in the cb-TENS group. However, there was no statistical significance in some analyses, which is presumed to be due to the insufficient number of rats. Lastly, we evaluated the functional recovery with video analysis, but objective evaluation using electromyography would be better to demonstrate reinnervation into the muscle more clearly, and further study is needed focused on this topic.

## Conclusion

In summary, cb-TENS can effectively facilitate the regeneration of a FN that was physically damaged in a rodent model. Facial function recovered more rapidly in the groups that received cb-TENS, which was confirmed through molecular markers and histological image analysis of the FNs. Although additional preclinical research is needed to prove safety and effectiveness, cb-TENS is an effective way to deliver a painless, non-invasive electrical stimulation with minimal side effects. Once long-term stability has been established, cb-TENS may help promote nerve regeneration and effective rehabilitation in patients with FNP.

## Materials and methods

### Animals

All experimental procedures and protocols were approved by the Institutional Animal Care and Use Committee of Samsung Medical Center. All experiments were performed under the guide for the care and use of laboratory animals. Sixty-six male Sprague Dawley (SD) rats (180–200 g) were used in this study. The estimated sample size was computed based on the previous study^37^. The sample size was calculated as sixteen rats per group when the significance level was 0.0125, and the power was 0.9. Considering 30% of drop-out rate due to various causes during the experiment, at least twenty-one rats were needed in each group, and finally, the study was conducted with twenty-two rats per group. Also, this study was carried out in compliance with the Animal Research: Reporting of In Vivo Experiments (ARRIVE) guidelines. Sixty-six male Sprague Dawley (SD) rats (8 weeks old, 180–200 g) were used in this study. The SD rats were maintained on a 12-h light–dark cycle with ad libitum access to food and water.

### Surgical procedure

All rats were anesthetized with 5% isoflurane in 1 L/min oxygen initially and with 1–3% isoflurane during the surgery. The face skin was shaved and cleansed with water and alcohol before surgery. The shaved area was larger than the patch electrode used in the cb-TENS stimulation groups. A 2.5-cm incision was made to expose the FN trunk of the preauricular area. The main trunk of the left FN was surgically cut 10 mm distally from the stylomastoid foramen. The nerve was immediately repaired with one 9-0 nylon epineural suture. The wound was closed, and the animal recovered from anesthesia in a cage. Mouth drooping and a loss of whisker orientation on the left side were indicators showing the recovery from anesthesia. The intact FN on the right side was used as an internal control to evaluate the facial dysfunction of the left side.

### Experimental groups and electrical stimulation

**Figure 6:**
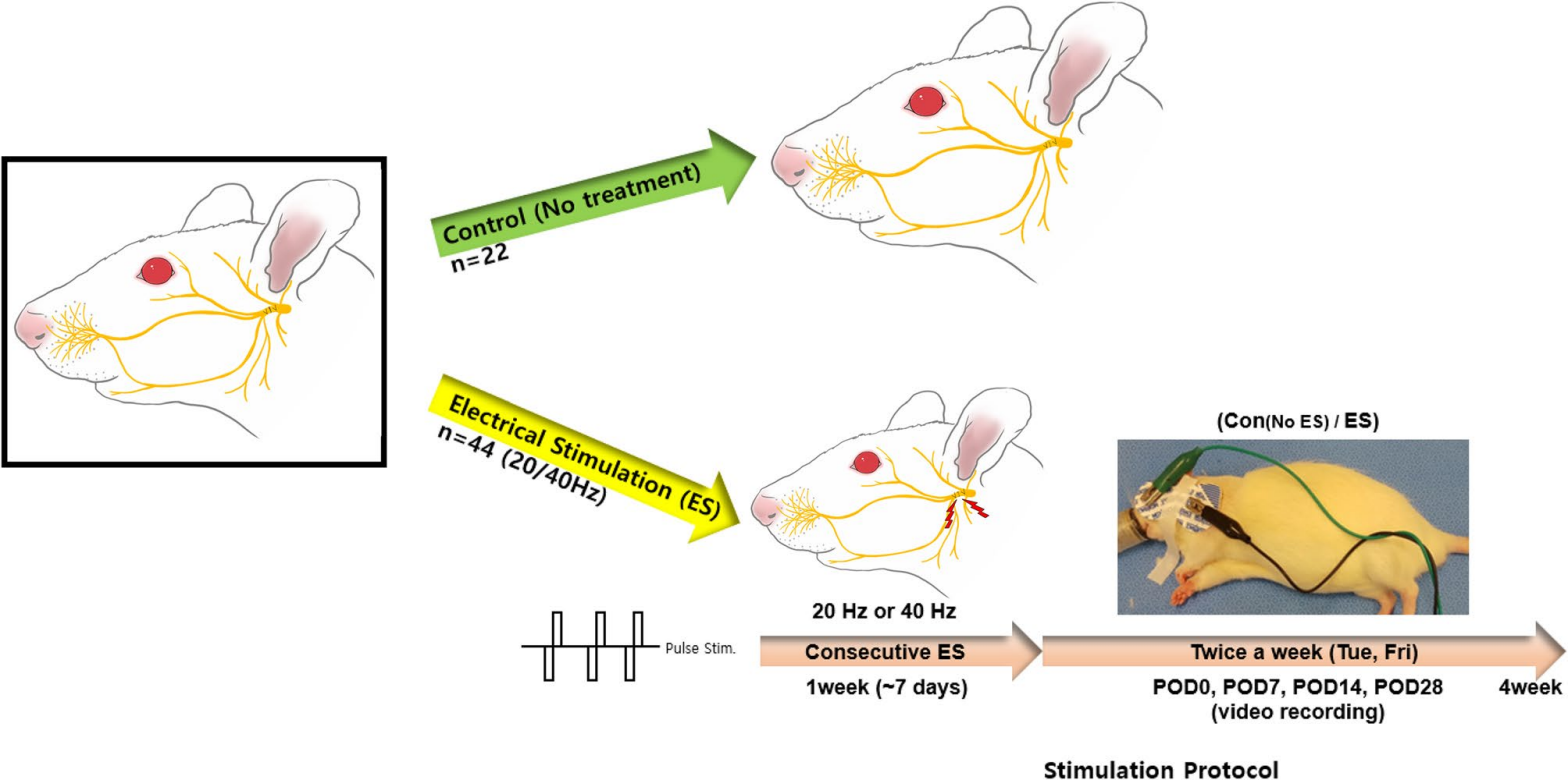
Experimental protocol. A total of 66 rats were divided into three groups: control (22 rats), 20 Hz (22 rats), and 40 Hz (22 rats). Charge-balanced Transcutaneous electrical nerve stimulation was performed daily for the first seven days and twice a week thereafter.

The rats were divided into three groups in this study: control, 20 Hz, and 40 Hz. The control group (22 rats) underwent surgery but did not receive any TENS treatments. The 20 Hz and 40 Hz groups had 22 rats each that underwent surgery and received the cb-TENS treatment with a pulse frequency of 20 Hz or 40 Hz, respectively (Fig. 6). The 20 Hz and 40 Hz groups received cb-TENS for 30 min daily from the day of the FN injury to post-operation day (POD) six-under gas anesthesia. From POD 7 to POD 28, cb-TENS was applied for 30 min twice a week. Ag/AgCl single snap electrodes of Kendal Meditrace (Meditrace, Covidien, MA, USA) were used in this study. The distance between the electrodes is slightly different depending on the size of the rat, but it was placed just behind the retro-auricular area where the FN trunk locates and in the distal portion as close as possible to the whisker. ES (20 or 40 Hz, ± 1 mA) was applied via a customized electrical stimulator (TPD-NH1, NuEyne, Seoul, Korea).

### Charge-balanced electrical stimulation waveform

The biphasic electric pulse signal used in this study was configured to have positive and negative current pulse duration and zero-current duration. The length of the positive and negative pulse duration times was 250 μsec. The interphase interval within one pulse was 5 μsec.

**Figure 7:**
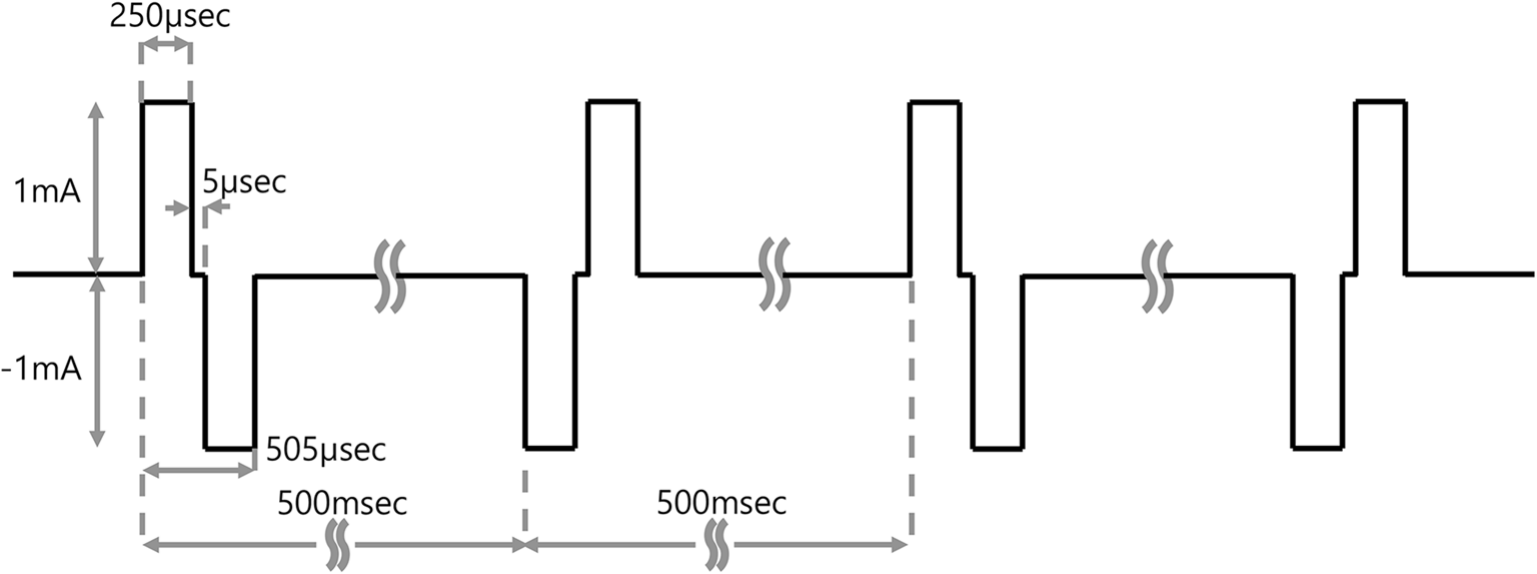
A pattern of charge-balanced electrical stimulation. A biphasic electric pulse signal was used that had positive and negative current pulse durations and a zero-current duration. After the first biphasic electric pulse signal with a positive current was presented, a zero-current duration of 5 μsec followed. Then, a negative current pulse occurred, followed by the second time with zero current.

Longer zero-current durations (25 ms for the 40-Hz stimulus and 50 ms for the 20-Hz stimulation) were appended after each application of the biphasic pulse according to each frequency condition. The magnitude of the absolute value of the positive current phase was 1 mA and equaled that of the negative current phase. After the zero-current duration, the next pulse was applied with an inverted waveform (Fig. 7).

### Functional recovery assessment

Whisker movement was measured at PODs 0, 7, 14, 21 and 28. The movement was recorded over a 2-min time frame by placing a rat without anesthesia in a cylinder-shaped cage in which the rat could only extend its head. The facial function was evaluated using the whisker movement according to the following scale: a grade of 0 for no detectable movement, 1 for detectable motion, 2 for significant but asymmetric voluntary motion, and 3 for symmetric voluntary motion^38,39^. The movement was scored at each time point by three blinded observers.

### Molecular analysis

The rats were euthanized by carbon dioxide asphyxiation on PODs 7, 14, and 28, and each FN was harvested. Total RNA was extracted by Trizol reagent (Invitrogen, Carlsbad, CA) and cDNA was synthesized from a respective RNA sample using Hybrid-R (305-101, GeneAll Biotechnology, Seoul, Korea). Real-time quantitative PCR (Quant Studio 6 Flex, Thermo Fisher Scientific) was performed with SYBR Green PCR Master (Applied Biosystems, 4309155). Forty cycles of the protocol were performed for 10 min at 25 °C, 120 min at 37 °C, and five minutes at 85 °C. The primer sequences were set for rat nerve growth factor (NGF, F: ACCTCTTCGGACACTCTGGA, R: TCCAACCCACACACTGACAC), rat brain-derived neurotrophic factor (BDNF, F: GGCGGCAGACAAAAAGACTG, R: ATGCCTTTTGTCTATGCCCC), rat vascular endothelial growth factor (VEGF, F: ACGGCAACCTCTCCAACTTC, R: CTGTCGGTGCTTCCAAGTCT), rat leukemia inhibitory factor (LIF, F: ATGAAGGTCTTGGCCACAGG, R: GTATGGCGCAGGTGGCATT), rat hepatocyte growth factor (HGF, F: GGAGGCAGCTATAAGGGGACA, R: CTTTACCGCGATAGCTCGAAG), rat glial cell-derived neurotrophic factor (GDNF, F: ATTCCAGAGGGAAAGGTCGC, R: GCTTCACAGGAACCGCTACA), rat interleukin-1β (IL-1β, F: AGTGTGTGATGTTCCCATTAG, R: GCTTATGTTCTGTCCATTGAG), rat IL-6 (F: TGTATGAACAGCGATGATG, R: AGAAGACCAGAGCAGATT), rat tumor necrosis factor-α (TNF-α, F: TTACGACGACCGTCAACTCG, R: TGGCCAATGGGTATGTCCAC), and rat transforming growth factor-β (TGF-β, F: ATGGTCAACGGCTCTCACAG, R: TGTGGGTGTTGGTAGACTGC).

### Transmission electron microscopy

Transmission electron microscopy (TEM) was performed to observe nerve fiber structures including myelin and axons using a JEM-1011 (JEOL, Tokyo, Japan) equipped with Camera-Megaview (Soft Imaging System, Münster, Germany). The harvested FN was fixed in 2% glutaraldehyde and 2% paraformaldehyde in 0.1 M phosphate buffer (pH 7.4) for 12 h and then washed in 0.1 M phosphate buffer. After post-fixing with 1% osmium tetroxide (OsO_4_) in phosphate buffer for two hours, the nerves were dehydrated in a gradient concentration of ethanol ranging from 50 to 100%, infiltrated with propylene oxide for 10 min, and embedded with a Poly/Bed 812 kit (Polysciences). Ultrathin sections of 80-nm thickness were obtained near the distal portion of the injured site. The samples were then placed on copper grids and stained twice with 3% uranyl acetate for 30 min and once with 3% lead citrate for 7 min. Morphological differences in the nerve fibers of each group were identified in TEM images.

### Immunofluorescence staining (IF) and confocal microscopy

An area 5 mm distal to the surgical site was selected for FN image analysis. The 5-mm-thick FN samples were dehydrated, embossed in a paraffin block, and transversely cut into 4-μm-thick sections using a microtome and a diamond knife. The sectioned samples were then laid on the slide glasses. The slides were blocked with Serum-Free Ready-To-Use (X0909) for 30 min and detected with the primary mouse monoclonal anti-neurofilament 200 (NF200, 1:200, Abcam, ab19386), rabbit monoclonal anti-S100 beta (S100B) (1:500, Abcam, ab52642), and the fluorescence-labeled secondary antibody. Then, the slides were stained with DAPI. The positive neurofilament 200 (NF200) staining signals were detected in each group with LSM780 with Axio Observer, a confocal laser scanning microscope (ZEISS, Germany). The area (μm^2^) of the positively stained cells in the corresponding field area was quantified using ImageJ software (National Institutes of Health, Bethesda, MD, USA). The area ratio of the positively-stained cells to the entire field was used as an indicator for the relative expression level of NF200.

### Statistical analysis

SAS version 9.4 (SAS Institute Inc., Cary, NC, USA) was used for all statistical analyses. Shaprio-Wilk test was performed to check the normality, and the nonparametric analysis was performed because normality was not satisfied. The functional score of facial function recovery between groups at each time was compared using the Kruskal–Wallis H test, and Bonferroni's correction was performed for repeated measurements. Considering multiple comparison, *p*-value < 0.0125 was determined to be significant. The analysis of mRNA expression was completed using the one-way analysis of variance followed by Tukey’s test for all paired comparisons.
